# Supplementary material for: Structural characterization of a highly-potent V3-glycan broadly neutralizing antibody bound to natively-glycosylated HIV-1 envelope
Source: Nat Commun. 2018 Mar 28;9:1251. doi: 10.1038/s41467-018-03632-y (PMC5871869; doi:10.1038/s41467-018-03632-y)
Supplement: Supplementary file 1 — Supplementary Information(PDF 1601 kb) [file 41467_2018_3632_MOESM1_ESM.pdf]

Structural characterization of a highly-potent V3-glycan broadly neutralizing antibody bound to natively-glycosylated HIV-1 Envelope

Barnes CO, *et al*.

Supplementary Information includes 11 supporting figures and 1 supplementary table.

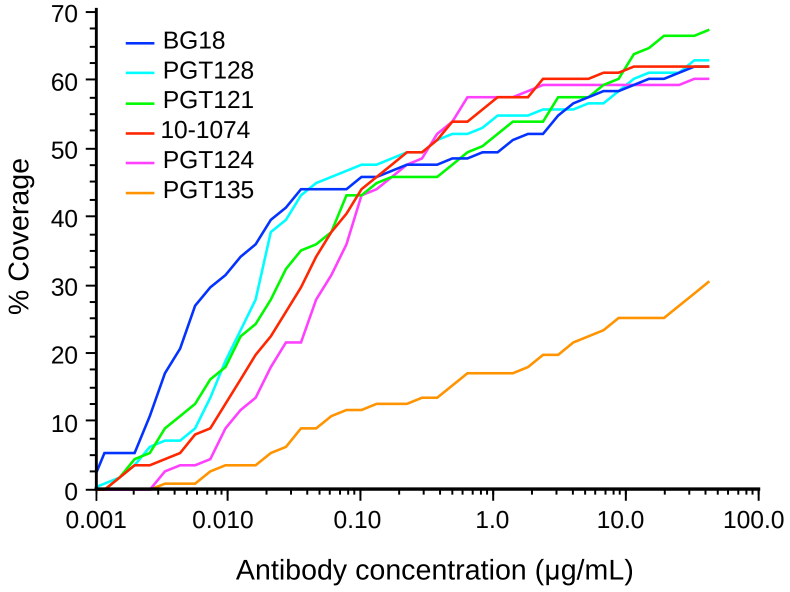

Supplementary Figure 1 | Neutralization coverage curves for selected V3/N332$_{gp120}$-glycan bNAbs. Coverage curves based on 107 common HIV-1 strains.

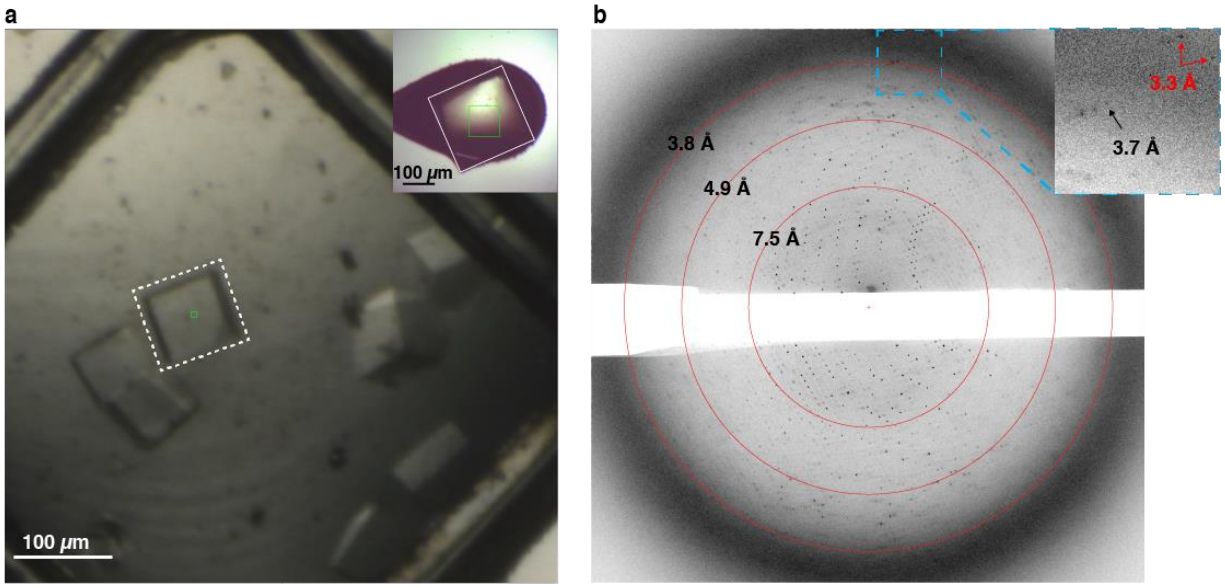

Supplementary Figure 2 | XFEL diffraction experiments of BG18-BG505-35O22 crystals. **(a)** BG18-BG505-35O22 cryo-cooled crystals mounted on specialized loops engineered for XFEL data collection at MFX (kind gift from the lab of Guillermo Calero, University of Pittsburgh; manuscript in preparation). Randomly oriented crystals had typical dimensions of ~75 µm x 75 µm x 50 µm, dimensions that were 2-3 fold smaller than crystals used to determine the 4.1 Å BG18-BG505-35O22 synchrotron structure (inset). Beam sizes are indicated by green boxes. **(b)** Representative XFEL diffraction image of BG18-BG505-35O22 crystals (panel **a**; dashed white box indicates crystal that was used to generate the diffraction image). The use of a 5 µm focused beam at MFX extended the resolution to 3.8 Å, with diffraction observed to 3.3 Å along the *c*-axis (inset). Comparable reflection intensities are typically reserved for larger Env-bNAb complex crystals (e.g., 600 µm x 100 µm x 100 µm for BG505-109L+3H-35O22 crystals[1], or 200 µm x 200 µm x 300 µm for BG505-IOMA-10-1074 crystals[2]) using a 50-100 µm focused synchrotron beam.

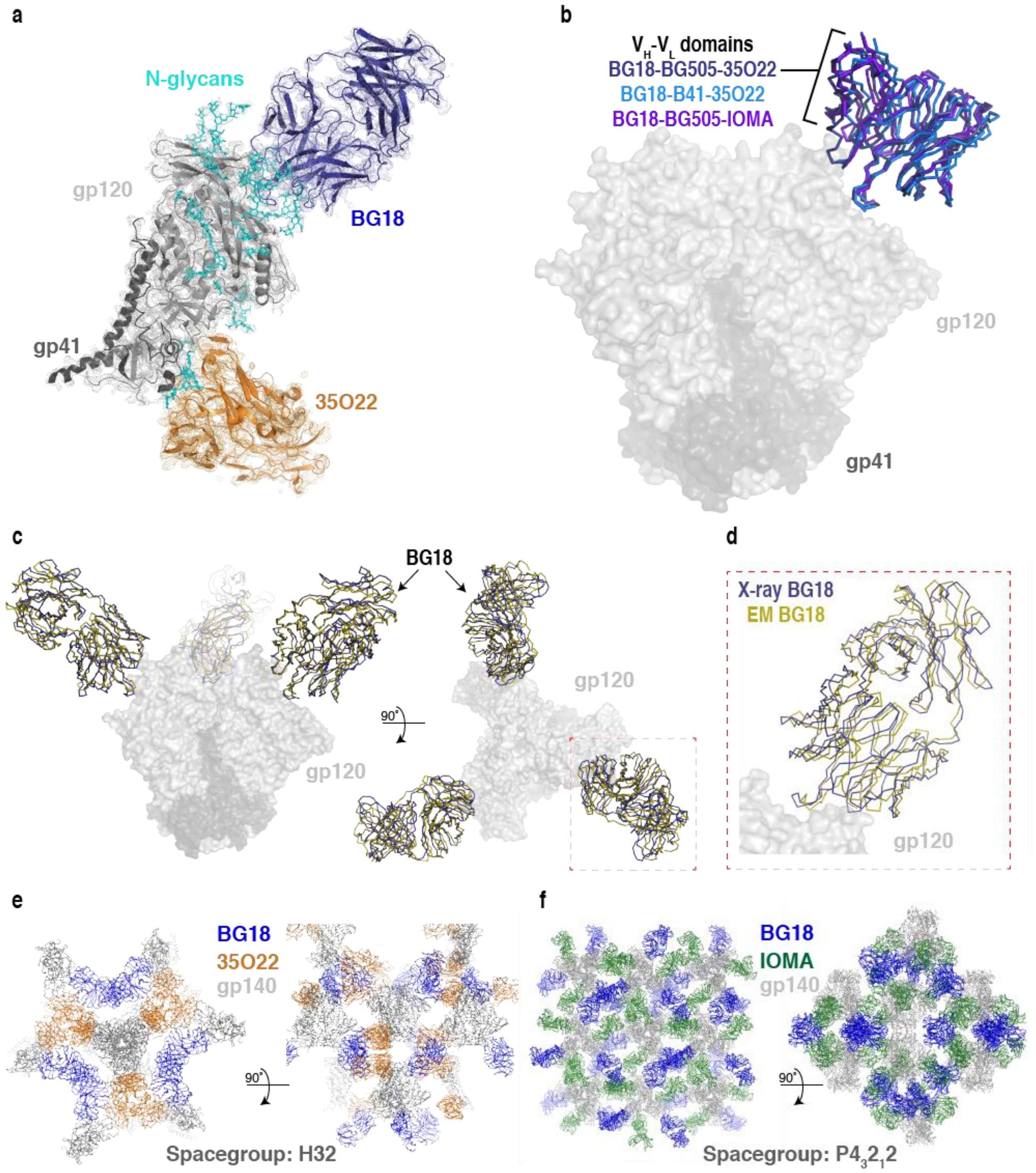

Supplementary Figure 3 | Comparison of Env-BG18 structures. (**a**) Cartoon representation of BG18-35O22-gp140 protomer. Electron density contoured at 1σ from 2F_{obs}-F_{calc} map of final refined model is shown. **(b)** Overlay of BG18 V_H-V_L domains from BG505-35O22-BG18 (dark blue), B41-35O22-BG18 (light blue), and BG505-IOMA-BG18 (purple) structures showed

similar orientations of BG18 V_H-V_L domains. **(c,d)** Overlay of BG18 Fabs from BG18-BG505-35O22 crystal structure with negative stain EM reconstruction of a BG18-BG505-179NC75 complex[3] demonstrating conservation of the common orientation adopted in the crystal structures. (**e,f**) Packing in crystals of complexes of BG18-BG505-35O22 and BG18-B41-35O22 **(e)** and BG18-BG505-IOMA **(f)**. Lattice contacts are formed by Fabs rather than Env trimer in BG18-BG505-35O22 and BG18-B41-35O22 structures, whereas both Fab and Env lattice contacts are observed in the BG18-BG505-IOMA structure.

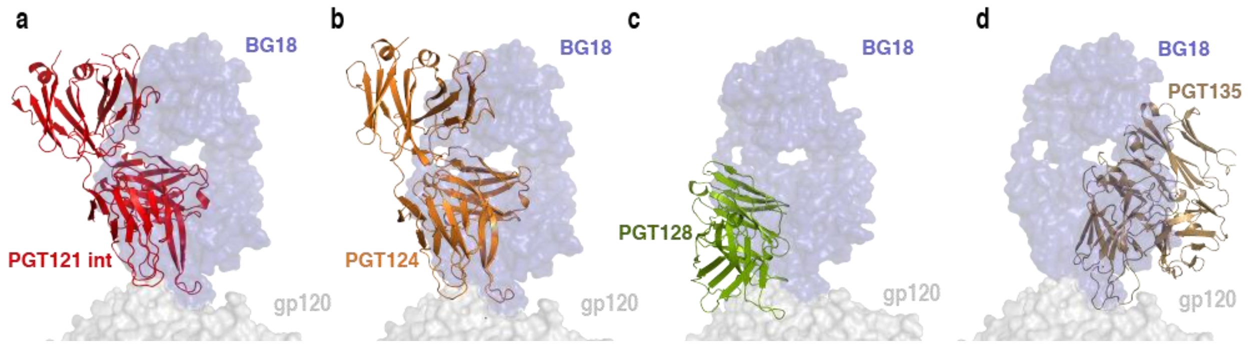

Supplementary Figure 4 | BG18 orientation compared to other V3/N332$_{gp120}$ glycan-targeting bNAbs. Comparison of BG18 (blue, surface) orientation for interacting with gp120 with (**a**) PGT121 immature precursor (red, PDB 5CEZ) (complexed with Env trimer), (**b**) PGT124 (light orange, PDB 4R2G) (complexed with Env trimer), (**c**) PGT128 (green, PDB 5ACO) (complexed with Env trimer), and (**d**) PGT135 (sand, PDB 4JM2) (complexed with monomeric gp120).

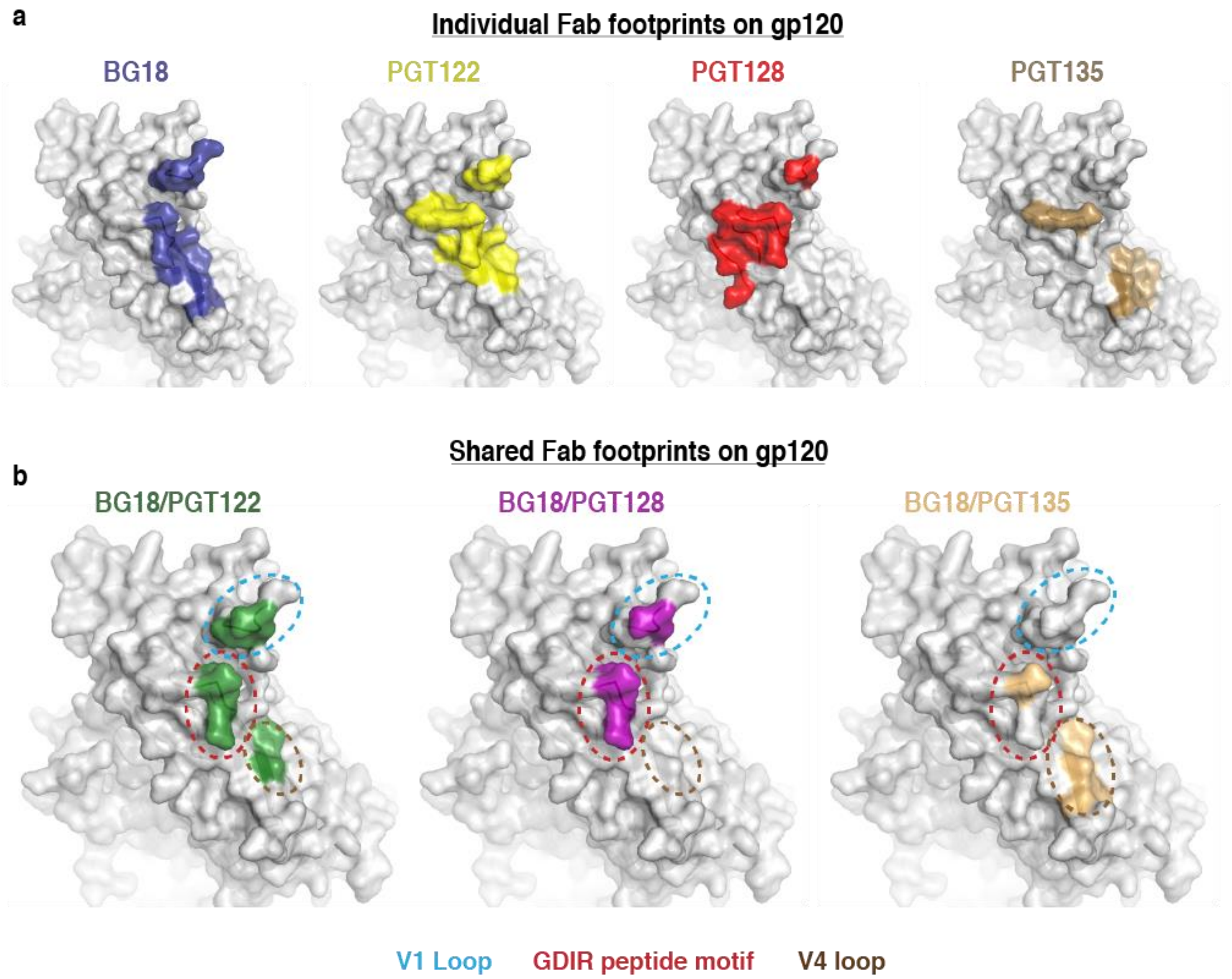

Supplementary Figure 5 | Comparison of select V3/N332$_{gp120}$-glycan bNAb footprints on the surface of gp120. (a) Protein epitopes on gp120 (surface representation) were defined as gp120 protein residues whose Cα atom was within 7 Å of Cα atoms on the bound Fab for BG18 (blue), PGT121-124 class (yellow, PGT122 shown), PGT128 (red) and PGT135 (sand). **(b)** Overlapping protein epitopes are indicated for BG18/PGT122 (green), BG18/PGT128 (purple), and BG18/PGT135 (light orange), with gp120 protein motifs highlighted (cyan – V1 loop, red – GDIR, brown – V4 loop).

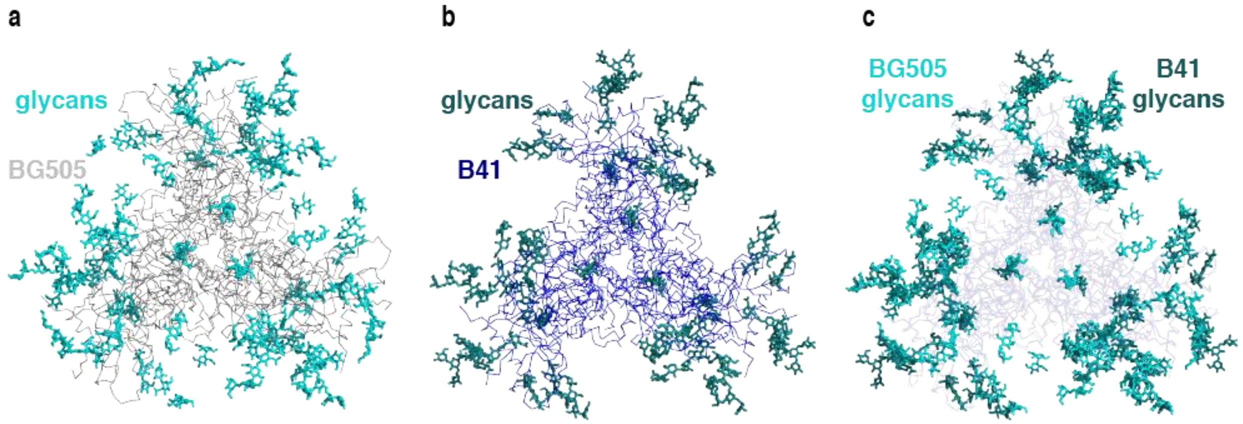

Supplementary Figure 6 | Natively-glycosylated Env trimers from clade A and clade B strains. (a-c) Ribbon representation of Env with modeled glycans showed as sticks for **(a)** BG505 (protein in gray; glycans in cyan), **(b)** B41 (protein blue; glycans teal), and **(c)** superposition of Env trimers (Env, blue gray; BG505 glycans, cyan; B41 glycans, teal). Despite resolution limitations that necessitated glycan modeling as predominantly oligomannose glycoforms in our BG18-B41 structure, the HIV-1 glycan shield was conserved across strains, extending 20-25 Å above the Env surface. In addition, the use of natively-glycosylated Env trimers in the crystallization complexes ensured accuracy of bNAb binding orientations and interfaces with Env trimer.

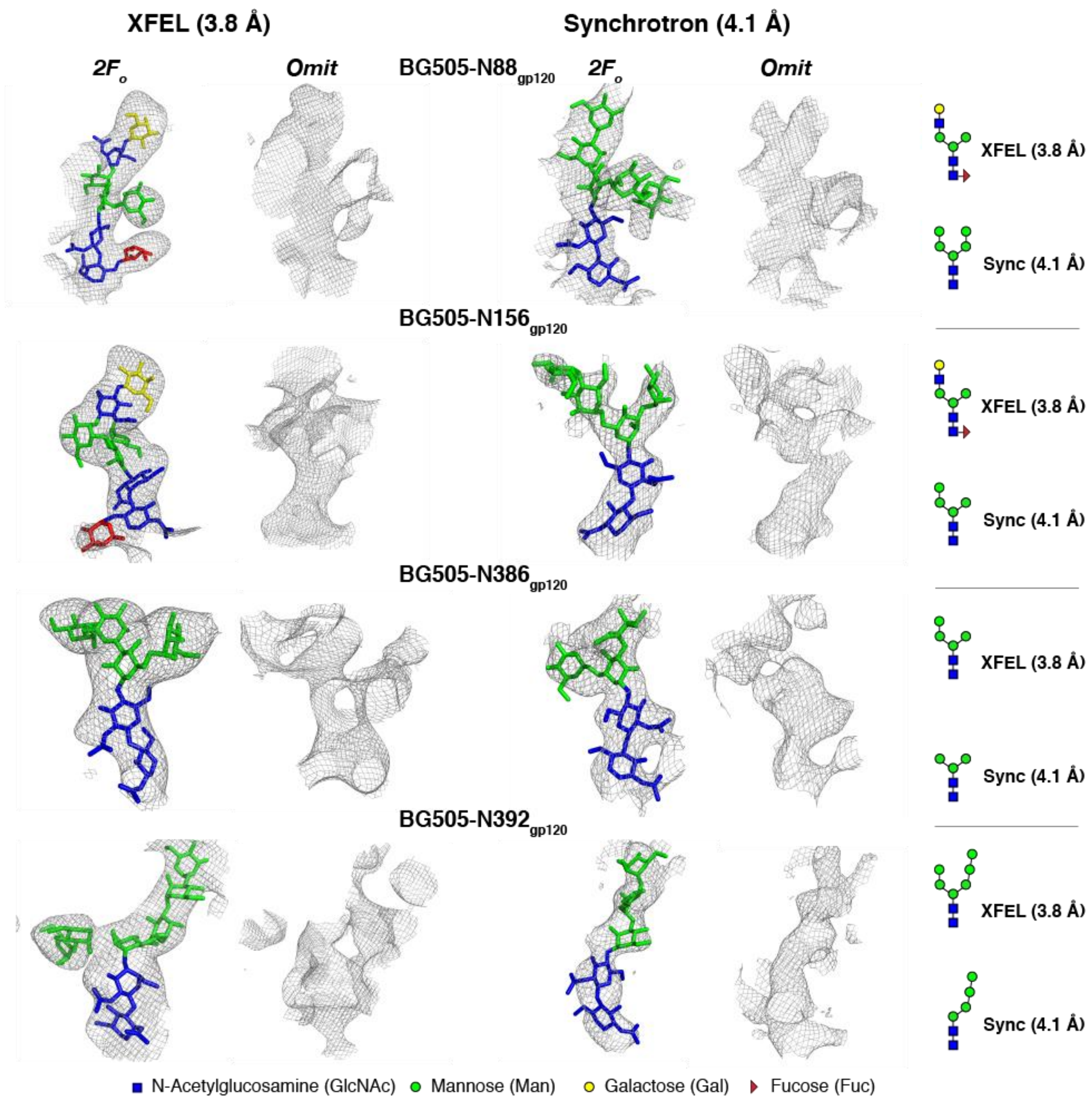

Supplementary Figure 7 | Comparison of glycans at individual BG505 PNGSs. Stick representation of glycans modeled in our BG18-BG505-35O22 structures (left – XFEL, right – synchrotron; blue, *N*-acetyl glucosamine; green, mannose; red, fucose; yellow, galactose). Electron density contoured at 1σ from $2F_{obs}$-F_{calc} maps calculated with model phases (2Fo column) or composite annealed omit maps calculated with phases in which the model was

omitted to reduce bias[4] (Omit column) are shown. Comprehensive tables comparing glycan forms seen by crystallography, cryo-EM, and by mass spectroscopy experiments[5] for individual PNGSs are shown in Supplemental Figures 5-8 in Gristick, et al[2]. Complex-type *N*-glycans observed in our XFEL structure for positions $N88_{gp120}$ and $N156_{gp120}$ were primarily identified through the presence of electron density for a core fucose residue, which when modeled resulted in a slight reduction of R_{free}. Residue-level schematic of modeled glycans in BG18-BG505-35O22 XFEL (top) and synchrotron (bottom) structures are illustrated on the far right (blue boxes, N-acetyl glucosamine; green circles, mannose; red triangle, fucose; yellow circles, galactose).

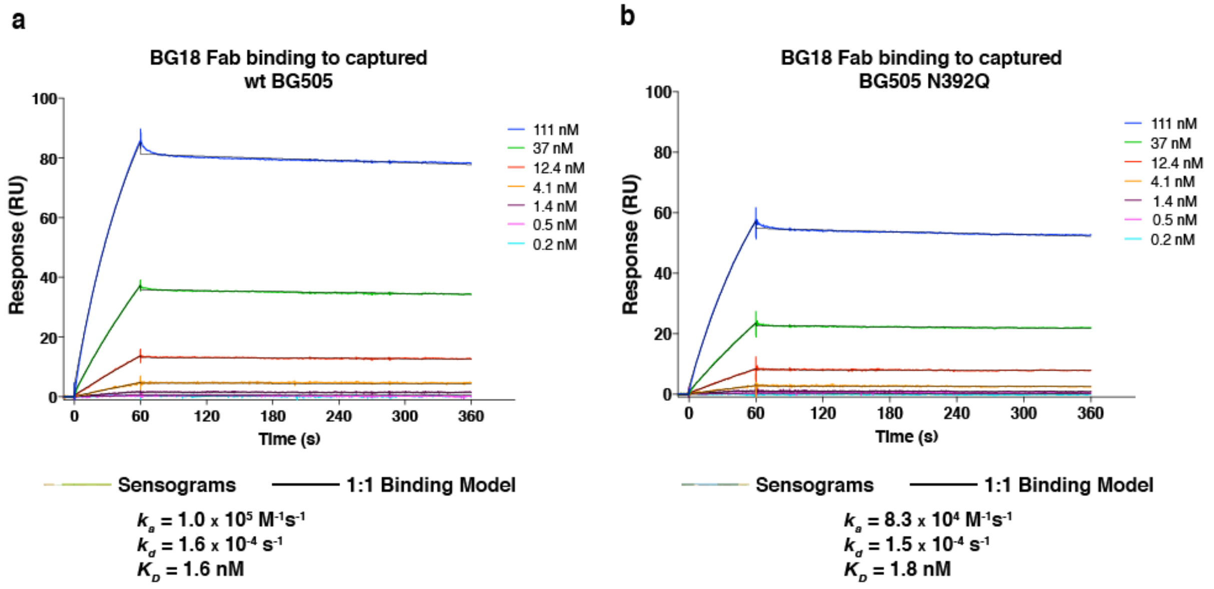

Supplementary Figure 8 | SPR binding assays of WT and N392Q BG505 with BG18.

Representative sensograms (multi-color), fits (black), K_D, k_a, and k_d values for binding of BG18 mature Fab to **(a)** wild-type BG505 SOSIP.664 and to **(b)** N392Q BG505 SOSIP.664. SOSIP trimers were captured on a protein A biosensor chip that was coupled with 8ANC195 IgG, and BG18 was flowed over the chip as a 3-fold dilution series with top concentrations of 111 nM (n=2).

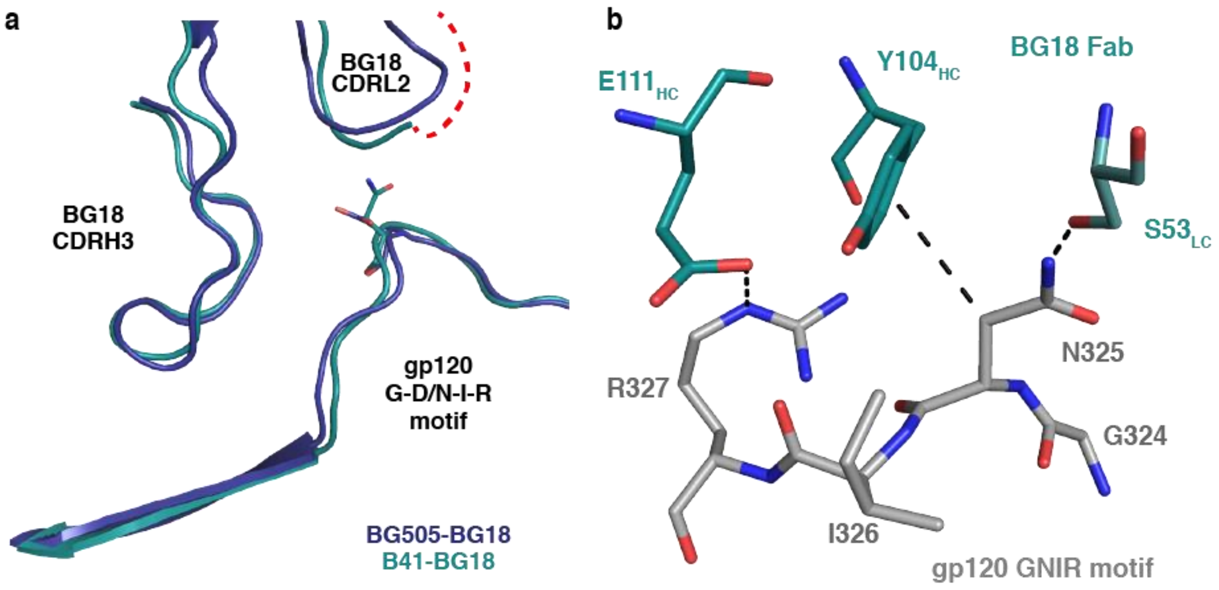

Supplementary Figure 9 | GNIR peptide motif interactions in BG18-B41 complex. (a) Cartoon representation of the overlay of BG18-BG505 (dark blue) and BG18-B41 (teal) after alignment of gp120s demonstrating a conserved architecture of BG18 binding to Env trimers from the BG505 and B41 strains. The dashed red line indicates disordered residues of CDRL2 in the BG18-B41 structure. **(b)** Stick representation of potential interactions between BG18 (teal) and the B41 gp120 GNIR motif (gray). Potential H-bonds are depicted as dashed lines.

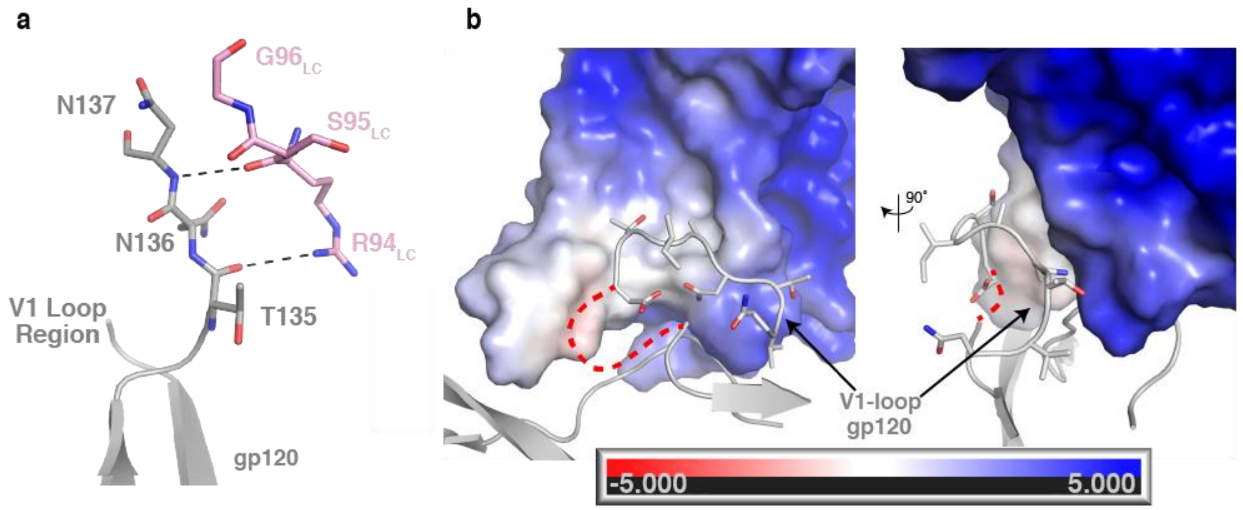

Supplementary Figure 10 | 10-1074 interactions with gp120 V1-loop. Analysis of a 10-1074–BG18 complex structure[2] (PDB 5T3Z) revealed minimal contact by 10-1074 with the gp120 V1-loop. **(a)** Cartoon and stick representation of 10-1074 CDRL3 residues (light pink) and the gp120 V1-loop (gray) revealed that only $R94_{LC}$ engages the V1-loop. **(b)** Electrostatic surface potentials with red indicating negative electrostatic potential and blue indicating positive electrostatic potential for 10-1074 shown with cartoon and stick representation of nearby gp120 elements. Structural analysis revealed that 10-1074 does not form a positively-charged cleft in the vicinity of the gp120 V1-loop (as seen in the BG18-BG505 complex structure), as the 10-1074 V_H domain sits ~25-30Å above the V1-loop elements, and Fab region in this area is neutral rather than positively-charged.

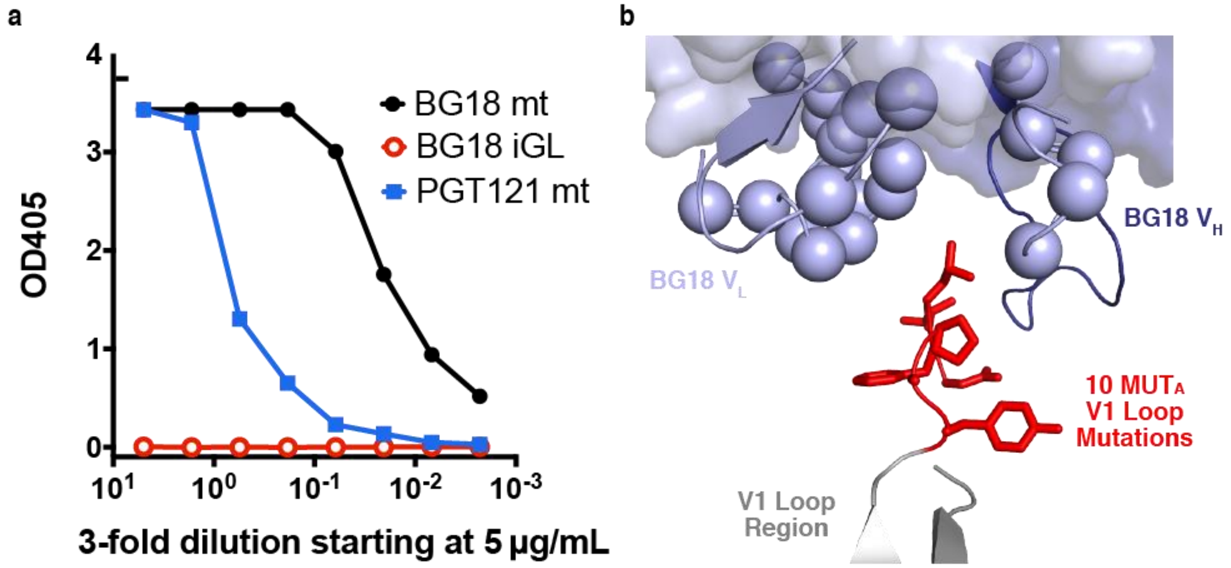

Supplementary Figure 11 | iGL BG18 interactions with SOSIP modified trimers. (a) ELISA demonstrating that the iGL of BG18 does not show detectable binding to 11MUT$_B$, a BG505 SOSIP-based immunogen designed to bind PGT121/10-1074 iGLs[6]. ELISA curves show the binding of the mature BG18 IgG (BG18 mt), the inferred germline BG18 IgG (iGL BG18), and the mature PGT121 IgG (PGT121 mt) to 11MUT$_B$. IgGs were evaluated in 3-fold dilutions starting at 5µg/mL as the highest concentration. **(b)** Modeling of BG18 Fab onto 10MUT$_A$ – MD39 (PDB 5T3S)[6], a modified BG505 SOSIP-based immunogen, illustrating how the iGL BG18 light chain CDR loop residues (Cαs represented as light blue spheres) lie in close proximity to mutated V1 loop residues that are necessary for iGL PGT121 binding (red sticks; mutated residues are conserved in 11MUT$_B$). The high degree of mutations in iGL BG18 relative to mature BG18 in this region rationalizes the lack of binding observed in panel **a**.

Supplementary Table 1. Primer sequences for site-directed mutagenesis of BG505 SOSIP.664

Primer Name	Primer Sequence (5´ to 3´)
BG505 N392A Forward	GCAATACATCAGGCCTGTTCCAGTCTACATGGATCTCAAATAC
BG505 N392A Reverse	GTATTTGAGATCCATGTAGACTGGAACAGGCCTGATGTATTGC

Supplementary References

1. Zhou, T. *et al.* Quantification of the Impact of the HIV-1-Glycan Shield on Antibody Elicitation. *Cell Rep* **19,** 719–732 (2017).
2. Gristick, H. B. *et al.* Natively glycosylated HIV-1 Env structure reveals new mode for antibody recognition of the CD4-binding site. *Nat. Struct. Mol. Biol.* **23,** 906–915 (2016).
3. Freund, N. T. *et al.* Coexistence of potent HIV-1 broadly neutralizing antibodies and antibody-sensitive viruses in a viremic controller. *Science Translational Medicine* **9,** (2017).
4. Adams, P. D. *et al.* PHENIX: a comprehensive Python-based system for macromolecular structure solution. *Acta Crystallogr. D Biol. Crystallogr.* **66,** 213–221 (2010).
5. Behrens, A.-J. *et al.* Composition and Antigenic Effects of Individual Glycan Sites of a Trimeric HIV-1 Envelope Glycoprotein. *Cell Rep* **14,** 2695–2706 (2016).
6. Steichen, J. M. *et al.* HIV Vaccine Design to Target Germline Precursors of Glycan-Dependent Broadly Neutralizing Antibodies. *Immunity* **45,** 483–496 (2016).
