# Supplementary material for: Structural characterization of a highly-potent V3-glycan broadly neutralizing antibody bound to natively-glycosylated HIV-1 envelope
Source: Nat Commun. 2018 Mar 28;9:1251. doi: 10.1038/s41467-018-03632-y (PMC5871869; doi:10.1038/s41467-018-03632-y)
Supplement: Supplementary file 3 — Description of Additional Supplementary Files(PDF 162 kb) [file 41467_2018_3632_MOESM3_ESM.pdf]

Description of Additional Supplementary Files

Supplementary Data 1. Comparison of interface between BG505 SOSIP.664 and BG18 Fab (PDB 6CH7) or 10-1074 Fab (PDB 5T3X), shown for SOSIP chain G (gp120) and Fab chains H (heavy chain) and L (light chain).
